# Astrocytic A1/A2 paradigm participates in glycogen mobilization mediated neuroprotection on reperfusion injury after ischemic stroke

**DOI:** 10.1186/s12974-021-02284-y

**Published:** 2021-10-13

**Authors:** Haiyun Guo, Ze Fan, Shiquan Wang, Lina Ma, Jin Wang, Doutong Yu, Zhen Zhang, Lin Wu, Zhengwu Peng, Wenming Liu, Wugang Hou, Yanhui Cai

**Affiliations:** 1 Department of Anesthesiology and Perioperative Medicine, Xijing Hospital, Fourth Military Medical University, Xi’an, Shaanxi China; 2 Department of Psychiatry, Xijing Hospital, Fourth Military Medical University, Xi’an, Shaanxi China; 3 Department of Anesthesiology, School of Stomatology, Fourth Military Medical University, Xi’an, Shaanxi China

**Keywords:** Ischemia/reperfusion injury, Glycogen, Astrocyte, Neuroprotection, Glycogen mobilization

## Abstract

**Background:**

Astrocytic glycogen works as an essential energy reserve for surrounding neurons and is reported to accumulate excessively during cerebral ischemia/reperfusion (I/R) injury. Our previous study found that accumulated glycogen mobilization exhibits a neuroprotective effect against I/R damage. In addition, ischemia could transform astrocytes into A1-like (toxic) and A2-like (protective) subtypes. However, the underlying mechanism behind accumulated glycogen mobilization-mediated neuroprotection in cerebral reperfusion injury and its relationship with the astrocytic A1/A2 paradigm is unknown.

**Methods:**

Astrocytic glycogen phosphorylase, the rate-limiting enzyme in glycogen mobilization, was specifically overexpressed and knocked down in mice and in cultured astrocytes. The I/R injury was imitated using a middle cerebral artery occlusion/reperfusion model in mice and an oxygen–glucose deprivation/reoxygenation model in cultured cells. Alterations in A1-like and A2-like astrocytes and the expression of phosphorylated nuclear transcription factor-κB (NF-κB) and phosphorylated signal transducer and activator of transcription 3 (STAT3) were determined by RNA sequencing, immunofluorescence and immunoblotting. Metabolites, including glycogen, NADPH, glutathione and reactive oxygen species (ROS), were analyzed by biochemical analysis.

**Results:**

Here, we observed that astrocytic glycogen mobilization inhibited A1-like astrocytes and enhanced A2-like astrocytes after reperfusion in an experimental ischemic stroke model in vivo and in vitro. In addition, glycogen mobilization could enhance the production of NADPH and glutathione by the pentose phosphate pathway (PPP) and reduce ROS levels during reperfusion. NF-κB inhibition and STAT3 activation caused by a decrease in ROS levels were responsible for glycogen mobilization-induced A1-like and A2-like astrocyte transformation after I/R. The astrocytic A1/A2 paradigm is closely correlated with glycogen mobilization-mediated neuroprotection in cerebral reperfusion injury.

**Conclusions:**

Our data suggest that ROS-mediated NF-κB inhibition and STAT3 activation are the key pathways for glycogen mobilization-induced neuroprotection and provide a promising metabolic target for brain reperfusion injury in ischemic stroke.

**Supplementary Information:**

The online version contains supplementary material available at 10.1186/s12974-021-02284-y.

## Background

Ischemic stroke seriously threatens human health worldwide, and restoration of cerebral blood flow using endovascular thrombectomy or thrombolysis after ischemic stroke is the principal therapeutic method for salvaging cells in the penumbra region and reducing the infarct volume [1]. However, blood supply restoration inevitably aggravates brain damage and augments the risk of hemorrhagic transformation and edema, called ischemia/reperfusion (I/R) injury [2]. Therefore, new avenues for alleviating reperfusion injury are urgently needed for patients suffering from ischemic stroke.

The brain consumes 20% of the energy used by the body, while accounting for only 2% of its weight [3]. Glycogen works as a crucial energy reserve during cerebral blood occlusion, and its balance is dynamically maintained via glycogenolysis and glycogenesis [4]. Glycogenesis is mainly controlled by glycogen synthase (GS) [5], and glycogen phosphorylase (GP) determines glycogenolysis flux [6]. Glycogenolysis activation, also named glycogen mobilization, is very important for maintaining physiological functions in the brain, such as synaptic transmission and sleep [7, 8]. In addition, its degradation product, glucose-6-phosphate, can transform into antioxidant substrates, such as NADPH and glutathione via the pentose phosphate pathway (PPP) [9, 10]. Previous studies have suggested that astrocytic glycogen accumulates excessively after I/R [11–13]. Our team recently pointed out that glycogenolysis dysfunction, but not glycogenesis enhancement, is responsible for reperfusion-induced glycogen accumulation [14]. Furthermore, glycogenolysis restimulation protects neural cells against reperfusion injury in the penumbra [14]. However, little is known about the underlying mechanism behind glycogenolysis mobilization-mediated neuroprotective effects on reperfusion injury after stroke, which impedes its clinical therapeutic application.

Astrocytes are crucial trophic supporters for neighboring neurons, and most brain glycogen stores in astrocytes but not neurons [15]. Recent studies have suggested that astrocytes can be divided into A1-like and A2-like subtypes [16]. A1-like astrocytes secrete components of the complement cascade and are fatal to neurons, and A2-like astrocytes produce neurotrophic factors and are protective [16]. In addition, nuclear transcription factor-κB (NF-κB) activation enhances A1-like astrocyte formation, and signal transducer and activator of transcription 3 (STAT3) activation accelerates A2-like astrocyte formation [17, 18]. Reactive oxygen species (ROS) can activate NF-κB and inhibit STAT3 in peripheral diseases [19, 20]. The above lines of evidence raise the question of whether glycogen mobilization regulates the astrocytic A1/A2 paradigm to fight against cerebral reperfusion injury.

Here, we provide evidence that glycogen mobilization suppresses A1-like astrocyte formation and enhances A2-like astrocyte formation during cerebral reperfusion damage via ROS-mediated NF-κB suppression and STAT3 activation. In addition, the astrocytic A1/A2 paradigm participates in glycogen mobilization-mediated neuroprotection in cerebral reperfusion injury. Together, our findings reveal the mechanism of glycogen mobilization-induced astrocytic A1/A2 transformation and provide a promising metabolic target for alleviating the irreversible outcomes of reperfusion injury after ischemic stroke.

## Methods

### Animals

Four-week-old male C57BL/6J mice, embryonic day 14–15 female C57BL/6J mice and 1-day-old neonatal C57BL/6J pups were purchased from the Experimental Animal Center of the Fourth Military Medical University. Astrocyte-specific brain isoforms of GP knock-in mice were customized by Cyagen (Santa Clara, USA) and verified by immunofluorescence. The experiments involving mice were approved by the Animal Care Committee of the Fourth Military Medical University and were conducted following the ARRIVE guidelines. Mice were housed in groups of four with ad libitum access to water and food in standard breeding cages at 23 ± 1 °C. All experiments and data analyses were conducted by investigators who were blinded to the animal groups, which were determined by randomization.

### Primary astrocyte, neuron and microglia culture

Primary astrocytes and microglia were acquired from the brain tissues of 1–3-day-old neonatal C57BL/6J pups according to previous studies [21, 22]. Astrocytes and microglia were cultured in complete medium consisting of DMEM (Thermo Fisher Scientific, USA) with 15% fetal bovine serum (Thermo Fisher Scientific). Primary neurons were obtained from the brains of the embryos of embryonic day 14–15 female C57BL/6J mice as previously reported [23]. Neurobasal medium (Thermo Fisher Scientific) with 2% B-27 (Thermo Fisher Scientific) and 1% glutamine (Thermo Fisher Scientific) was used for primary neuron culture.

### Cell coculture model

A cell coculture system was established using hanging inserts with porous membranes (3 μm, Millipore, USA). The volume of the coculture medium was 400 μL, and the distance from the neuronal or microglial membrane to the astrocytic layer was 0.15 cm. The coculture medium was neurobasal medium with 15% fetal bovine serum, 1% glutamine and 2% B-27 for the astrocyte–neuron coculture system and DMEM with 15% fetal bovine serum for the astrocyte–microglia coculture system.

### Reagents

Lipopolysaccharide (LPS) was obtained from Thermo Fisher Scientific (No. 00-4976-93). The ROS inducer BAY 87-2243 (BAY) [24] was obtained from Sigma-Aldrich (SML2384, USA). The C3 level in the culture medium was determined with the Complement C3 Mouse ELISA Kit (Abcam, USA).

### I/R mouse model

The I/R mouse model was established using the middle cerebral artery occlusion/reperfusion (MCAO/R) model. Eight-week-old C57BL/6J male mice were anesthetized with 1.4% isoflurane. A small incision was made on the anterior aspect of the neck along the midline, and the muscle was removed under a surgical microscope. Next, a monofilament (RWD Life Science, China) was introduced into the internal carotid artery through a small incision and extended into the right middle cerebral artery of the operated mouse. The monofilament was withdrawn after 60 min, and the wound was sutured. A 37 °C heating pad was applied to keep the mouse body temperature dynamically controlled during the surgery. The sham group also underwent the surgical procedure as the MCAO group except for artery occlusion. The mouse frontal cortex from bregma to bregma—1 mm in the coronal plane was considered as the penumbra of the ischemic hemisphere following transient MCAO [25, 26].

### In vitro I/R model

An in vitro I/R model was imitated using an oxygen–glucose deprivation/reoxygenation (OGD/R) model. During OGD, the cell culture medium was glucose-free DMEM (Thermo Fisher Scientific) for astrocytes and glucose-free neurobasal medium (Thermo Fisher Scientific) for neurons. The cells were transferred into a humidified hermetic chamber (Billups-Rothenberg, USA) at 37 °C containing an anaerobic gas mixture (5% CO_2_ and 95% N_2_). The cells were removed from the chamber after 2 h of OGD, and the glucose-free DMEM was replaced with complete culture medium. The culture medium was also changed before and after OGD in the non-OGD group.

### Immunofluorescence

The mice were intraperitoneally injected with pentobarbital sodium (125 mg/kg, Sigma-Aldrich) and transcardially perfused with saline followed by paraformaldehyde (4%, Sigma-Aldrich). The cerebral tissues were then fixed with 4% paraformaldehyde for 24 h and then postfixed with 20% and 30% sucrose (Sigma-Aldrich) for 24 h separately. Next, the brain tissues were frozen, and ten coronal brain section (12 μm) from the area approximately bregma—0.5 mm were cut with a cryostat and collected on slides. The slides were incubated with primary antibodies for 12 h and with secondary antibodies for 2 h. The antibodies used for immunofluorescence are shown in Additional file 1: Table S1. All cells were stained with DAPI (GeneCopoeia, USA), and images were captured with a confocal microscope (Olympus, Japan). ImageJ software was used to analyze the fluorescence intensity.

### Immunoblotting

Immunoblotting was performed according to previous studies [27]. The antibodies used for immunoblotting are shown in Additional file 1: Table S1.

### Preparation and characterization of a polyclonal antibody targeting phosphorylated GP

An antibody against the phosphorylated brain isoform of GP (Ser14) is not commercially available. Thus, an antibody was synthesized by Genecreate Biological Engineering (China). In brief, a 14-amino acid phosphorylated peptide (TDSERQKQI-pS-VRGI) predicted by epitope analysis software was chemically synthesized as an immunogen and subcutaneously injected into male rabbits at 2-week intervals. The antiserum was harvested after 4 immunizations and subjected to phosphorylated affinity purification twice followed by six rounds of nonphosphorylated affinity purification. The dilution ratio of the synthesized phosphorylated antibody for specifically recognizing phosphorylated GP was verified using ELISA by Genecreate Biological Engineering.

### GP overexpression and CRISPR/Cas9-mediated knockdown in vitro

A lentivirus driving the expression of GP and a CRISPR/Cas9 lentivirus carrying an sgRNA (ATGCGACGAAGCCACTTATC) targeting the *gp* gene (GenBank: NM_153781) were constructed by GeneChem Ltd. (China). A blank vector without the *gp* gene and a lentivirus carrying scrambled sgRNA (CGCTTCCGCGGCCCGTTCAA) were constructed as negative controls separately. The lentiviruses were mixed with culture medium at a multiplicity of infection of 100 for 72 h for infection. GP overexpression and knockdown models in vitro were verified using immunoblotting.

### STAT3 knockdown in vitro

A lentivirus carrying an shRNA (TAACTTCAGACCCGTCAACAAATTCAAGAGATTTGTTGACGGGTCTGAAGTTTTTTTTC) targeting the *stat3* gene (GenBank: NM_213659) were constructed by GeneChem Ltd. (China). A blank vector carrying scrambled shRNA (GATCCCTGCATAGACCTGCTATCGTTCAAGACGCGATAGCAGGTCTATGCAGTTTTTTGTCGACA) were constructed as a negative control. The infection procedure was performed the same as that in GP knockdown model.

### Astrocyte-specific GP knockdown in rodents

An adeno-associated virus (AAV) carrying an astrocyte-specific gfaABC1D promoter [28] driving the expression of a 19-nucleotide (GGTGGCTGCTGTTGTGTAA) siRNA targeting the *gp* gene was purchased from GeneChem Ltd. Two microliters of AAV (5 × 10^12^ v.g./ml) was injected into the lateral ventricle 3 weeks before MCAO. The mice were anesthetized with 1.4% isoflurane through a facemask and placed in a stereotaxic head frame (Stoelting, USA). A 1.0-mm burr hole was made with a dental trephine drill (NSK Ltd., Japan) after the scalp was retracted. The coordinates for stereotaxic injection were as follows: 1.0 mm to the right of the midline, 0.22 mm posterior to bregma and 1.4 mm deep. A Hamilton syringe was used to infuse 2 μL of AAV into the lateral ventricle over 20 min. The needle was left in place to prevent reflux in the lateral ventricle for an additional 20 min, and the skull was sealed with quick self-curing acrylic resin (Yamahachi Dental Mfg., Japan). The scalp was sutured closed. An electronic thermostat-controlled warming blanket was used to maintain the body temperature of each mouse at 37 ± 0.5 °C during the experiments.

### Triphenyl tetrazolium chloride (TTC) staining

Brain tissues were continuously cut into coronal slices at 1 mm intervals and immersed in 2% TTC solution (MP Biomedicals, USA) at 37 °C for 20 min. The TTC-stained areas (red) indicate the nonischemic regions, and the white areas represent the ischemic regions. The lesion area was analyzed using ImageJ software. The infarct volume was calculated according to the formula: (area of the contralateral hemisphere—nonlesioned area in the ipsilateral hemisphere)/area of the contralateral hemisphere.

### Metabolic analysis of GP and GS enzyme activity

The GP and GS activities were determined according to our previous study [14]. Adherent astrocytes grew to 90% confluency and were dissociated with lysate buffer (GMS12054.2, Genmed Scientifics, China). The lysis time was 30 min on ice to ensure that the enzyme was fully dissolved in lysis buffer. Then, the lysate was collected in a prechilled microcentrifuge tube with a cell scraper and centrifuged at 13,000 rpm at 4 °C for 15 min. Finally, the supernatant was used as a source of the enzyme for evaluation.

Active GS activity was detected with a Glycogen Synthase Activity Assay Kit (GMS50500.1, Genmed Scientifics). A substrate solution containing uridine diphosphate glucose (UDPG), phosphoenolpyruvic acid and NADH was mixed. Then, an enzyme solution containing the supernatant of cell lysate, pyruvate kinase and lactate dehydrogenase was added to prepare a reaction solution. The UDPG transformed to uridine diphosphate (UDP) represented the activity of GS. Phosphoenolpyruvic acid and UDP as substrates then changed into pyruvate and UTP by pyruvate kinase, and pyruvate was converted to lactate by lactate dehydrogenase in parallel with the transformation from NADH to NAD^+^, reflected as the reduction in absorbance at 340 nm. The activity of GS was measured by subtracting the absorbance at 5 min from that at 0 min at 340 nm using a microplate reader (TECAN). The sample preparation was performed on ice, the enzyme reaction procedure was performed at 30 °C, and the results were normalized to the protein levels in homologous samples. The reaction formula is as follows:

$$\begin{gathered} {\text{UDPG}} + \left( {{\text{Glycogen}}} \right)_{n} \rightarrow ^{{{\text{GS}}}} \rightarrow{\text{UDP}} + \left( {{\text{Glycogen}}} \right)_{n + 1} \hfill \\ {\text{Phosphoenolpyruvate}} + {\text{UDP}}\rightarrow ^{{{\text{Pyruvate}}\,{\text{kinase}}}}\rightarrow {\text{Pyruvate}} + {\text{UTP}} \hfill \\ {\text{Pyruvate}} + {\text{NADH}}\rightarrow ^{{{\text{Lactate}}\,{\text{dehydrogenase}}}}\rightarrow {\text{Lactate}} + {\text{NAD}}^{ + } \hfill \\ \end{gathered}$$

Active GP activity was analyzed with a glycogen phosphorylase activity assay kit (GMS50092.1, Genmed Scientifics). The decomposition rate of glycogen to glucose-1-phosphate represented the activity of GP. With phosphoglucomutase and glucose-6-phosphate dehydrogenase, glycogen-derived glucose-1-phosphate was first transformed to glucose-6-phosphate, which was then converted into 6-phosphogluconate, and this procedure was combined with the conversion of NADP^+^ to NADPH, reflected as the increase in absorbance at 340 nm. The GP activity was measured by subtracting the absorbance at 0 min from that at 5 min at 340 nm using a microplate reader, and the results were normalized to the protein levels in homologous samples. The reaction formula is as follows:

$$\begin{gathered} {\text{Pi}} + \left( {{\text{Glycogen}}} \right)_{n} \rightarrow ^{{{\text{GP}}}}\rightarrow {\text{Glucose - }}1{\text{ - phosphate}} + \left( {{\text{Glycogen}}} \right)_{n - 1} \hfill \\ {\text{Glucose - }}1{\text{ - phosphate}}\rightarrow ^{{{\text{Phosphoglucomutase}}}}\rightarrow {\text{Glucose - }}6{\text{ - phosphate}} \hfill \\ {\text{Glucose - }}6{\text{ - phosphate}} + {\text{NADP}}^{ + } \rightarrow ^{{\text{Glucose - 6 - phosphate dehydrogenase}}}\rightarrow {\text{NADPH}} + 6{\text{ - phosphogluconate}} \hfill \\ \end{gathered}$$

### RNA sequencing

Total RNA in the cultured astrocytes was extracted using Trizol reagent kit (Invitrogen, USA) according to the manufacturer’s protocol. RNA quality was assessed on an Agilent 2100 Bioanalyzer (Agilent Technologies, USA) and checked using RNase free agarose gel electrophoresis. Then, the following RNA sequencing was performed by the Gene Denovo Biotechnology Co. (Guangzhou, China).

### Metabolic analysis of metabolite levels

The presence of various enzymes and proteins could interfere with the detection of metabolites in cultured astrocytes and consequently be removed using the Deproteinizing Sample Preparation Kit (Biovision, USA). A glycogen assay kit (Biovision) was used to detect glycogen levels in cultured astrocytes. ROS levels were determined using the ROS Detection Assay Kit (Biovision). A glucose-6-phosphate colorimetric assay kit (Biovision) was used to detect the glucose-6-phosphate levels. NADPH levels were determined with the NADPH Quantitation Fluorometric Assay Kit (Biovision). Glutathione levels were analyzed with the Glutathione Fluorometric Assay Kit (Biovision).

To determine the glycogen levels in the brain, the mice were decapitated, and brain tissues in the penumbra were immersed in liquid nitrogen. Then, the brain tissues (10 mg) were homogenized with 200 μL 30% KOH on ice, and the homogenates were boiled for 10 min to inactivate enzymes. The boiled samples were centrifuged at 12,000 rpm at 4 °C for 10 min to remove insoluble sediments. Then, the supernatant was ready for the assay using a glycogen assay kit (Biovision), and the results were normalized to the protein levels in homologous samples.

### Cell survival analysis

Cell Counting Kit-8 (CCK-8, Sigma-Aldrich) was used to detect the viability of astrocytes and neurons. An LDH-Cytotoxicity Colorimetric Assay Kit (Biovision) was used to analyze lactate dehydrogenase (LDH) release in the medium.

### Statistical analysis

All data are presented as the mean ± SD and were analyzed using SPSS 20.0 software (IBM). For the parametric analysis, the differences between two independent groups were analyzed using independent *t* tests. The differences among multiple groups were determined by one-way ANOVA. Two-way ANOVA was performed to determine differences between groups at multiple timepoints. Post hoc comparison was conducted according to the results of the test for equality of variance. Asymptotic two-sided *P* values less than 0.05 were considered significant.

## Results

### Glycogen mobilization suppresses A1-like astrocyte formation and enhances A2-like astrocyte formation during I/R

**Fig. 1:**
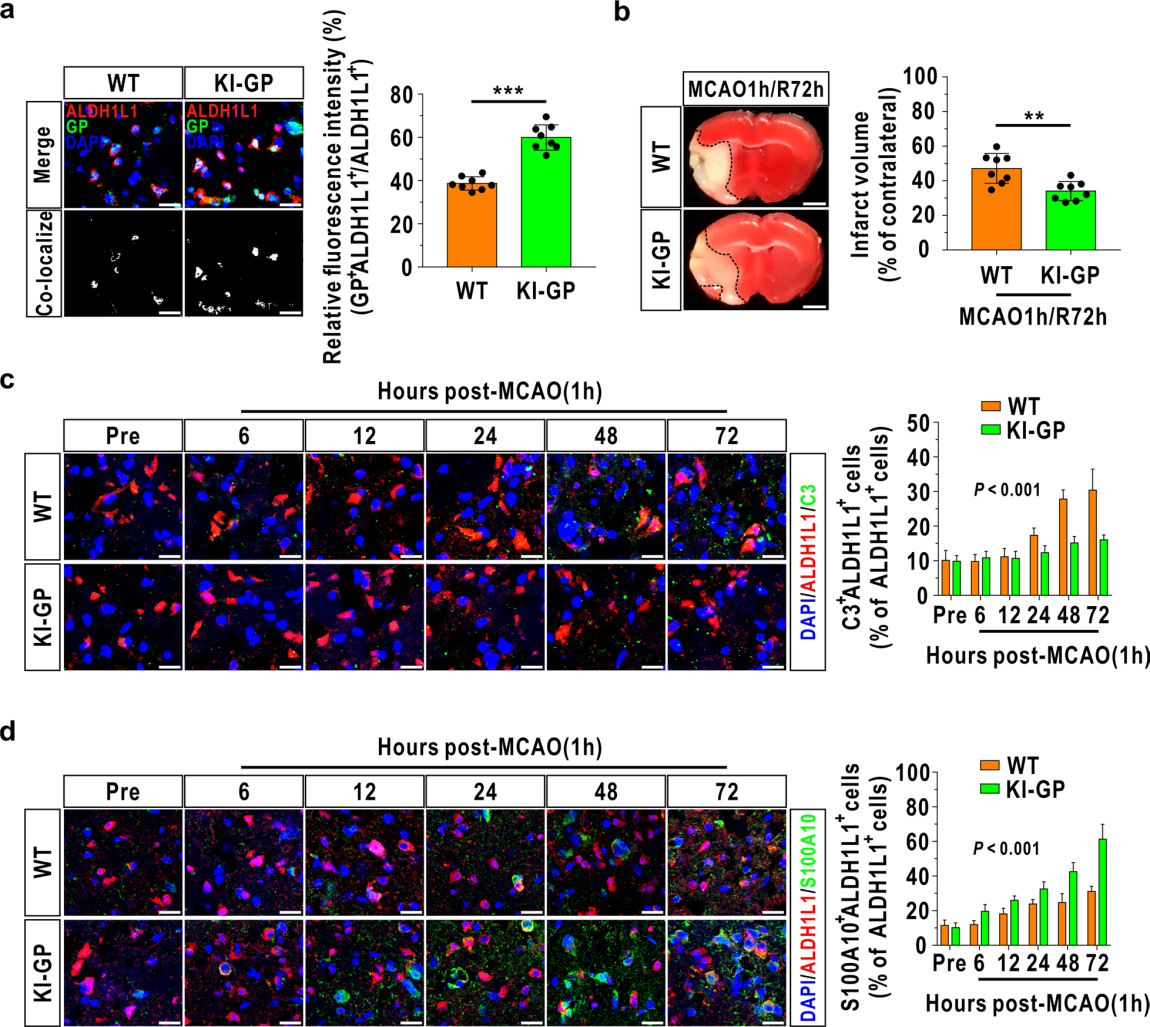
Glycogen mobilization suppresses A1-like astrocyte formation and accelerates A2-like astrocyte formation during MCAO/R. **a** Verification of successful generation of astrocyte-specific GP overexpression mice (KI-GP). Left panels: Representative images using immunofluorescence staining with antibodies against ALDH1L1 and GP in frontal cortex area 1 of the KI-GP mouse brain. The scale bars represent 20 μm. Right panels: Quantitative analysis of the relative fluorescence intensity of GP (*n* = 8). The relative fluorescence intensity was calculated by dividing the fluorescence intensity in the colocalization area by the fluorescence intensity in the ALDH1L1^+^ area. Astrocytes were marked with ALDH1L1. WT represents wild-type animals. **b** Left panel: Representative TTC-stained brain slices at 72 h following MCAO/R. The scale bars are 1 mm. Right panel: Quantitative analysis of the infarct volume in the left panel (*n* = 8). **c** Left panel: Coronal images of the cerebral ischemic penumbra following immunofluorescence analysis with antibodies against ALDH1L1 and C3 before (Pre) and after MCAO/R injury. The scale bars represent 20 μm. Right panel: Percentage of C3^+^ALDH1L1^+^ cells relative to ALDH1L1^+^ cells in the left panel (*n* = 8). **d** Left panel: Coronal images of the cerebral ischemic penumbra following immunofluorescence analysis with antibodies against ALDH1L1 and S100A10 before and after MCAO/R injury. The scale bars represent 20 μm. Right panel: Percentage of S100A10^+^ALDH1L1^+^ cells relative to ALDH1L1^+^ cells in the left panel (*n* = 8). **P < 0.01, ***P < 0.001. Independent *t* test for **a**, **b**. Two-way ANOVA for **c**, **d**

To uncover the relationship between glycogen mobilization and the astrocyte A1/A2 paradigm, we first used astrocyte-specific GP overexpression transgenic mice (KI-GP), which were constructed in our previous study [14], and verified by immunofluorescence using another astrocyte marker, ALDH1L1 [29] (Fig. 1a). Moreover, TTC staining indicated that astrocytic GP overexpression significantly decreased the infarct volume at 72 h after reperfusion (Fig. 1b). Previous evidence suggests that complement C3 is the key marker of A1-like astrocytes [16] and that S100A10 is the crucial identifier for A2-like astrocytes [30, 31]. Here, we used double-labeling immunofluorescence to specifically identify C3-positive astrocytes and S100A10-positive astrocytes in vivo and found that the percentage of A1-like astrocytes and A2-like astrocytes was increased and reached a peak at 72 h in wild-type (WT) mice after cerebrovascular recirculation (Fig. 1c, d). In addition, the KI-GP mice presented fewer A1-like astrocytes and more A2-like astrocytes than the WT mice during MCAO/R stress (Fig. 1c, d).

**Fig. 2:**
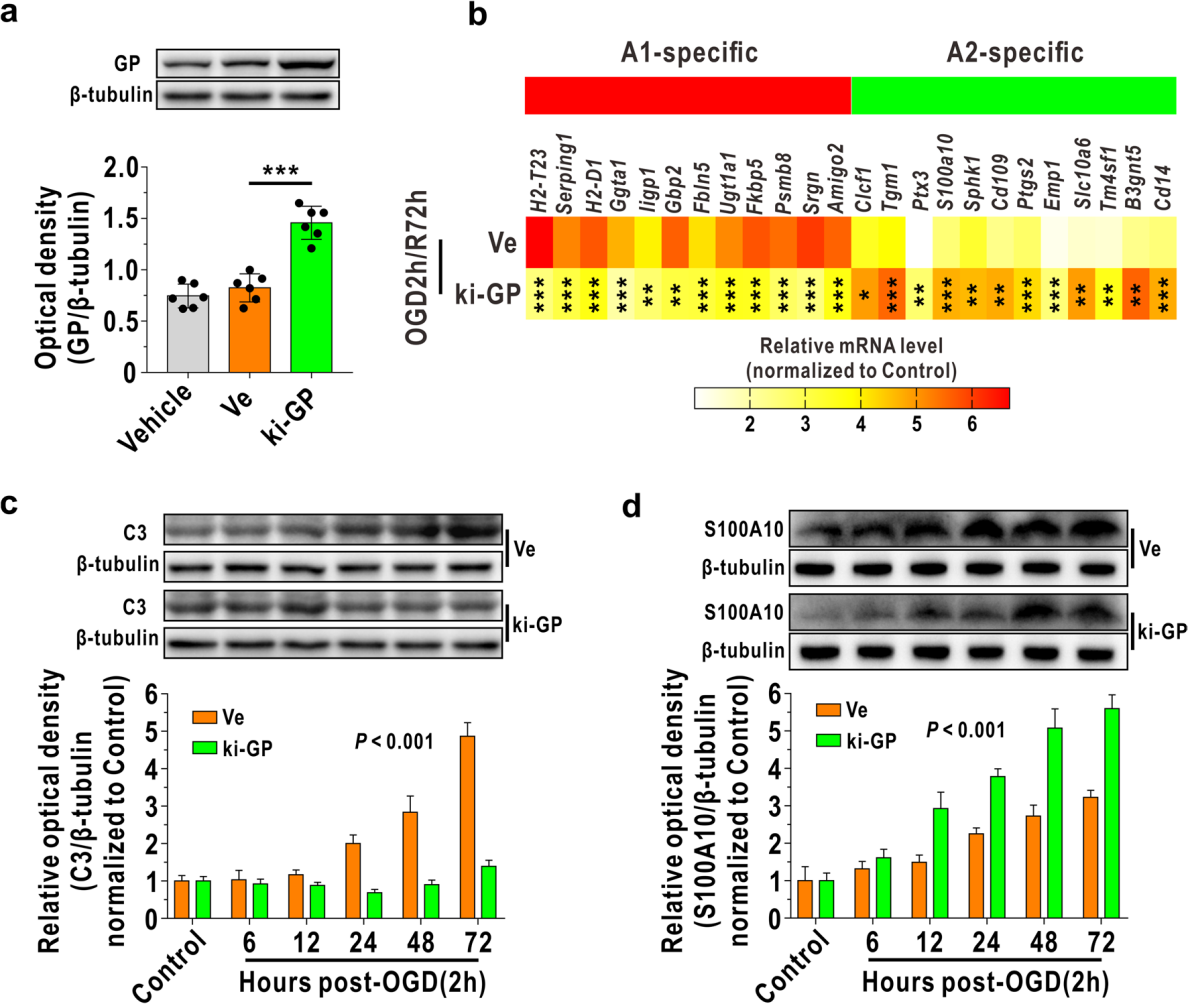
Glycogen mobilization suppresses A1-like astrocyte formation and accelerates A2-like astrocyte formation during OGD/R. **a** Verification of GP overexpression in cultured astrocytes (ki-GP) using immunoblotting (*n* = 6). Vehicle represents unaffected astrocytes. Ve represents astrocytes affected by blank lentiviruses. **b** Heat map of relative mRNA levels of 12 target genes of A1-like astrocyte and A2-like astrocyte at 72 h following reoxygenation, determined by RNA sequencing (*n* = 4). **c** Astrocytic C3 levels at different timepoints before and after OGD/R challenge, as measured by immunoblotting (*n* = 6). The relative optical density was calculated by dividing the density of the C3 band by that of the corresponding β-tubulin band and then normalizing to the control group. **d** Astrocytic S100A10 levels at different timepoints before and after OGD/R challenge, analyzed by immunoblotting (*n* = 6). *P < 0.05, **P < 0.01, ***P < 0.001. One-way ANOVA followed by LSD post hoc analysis for **a**. Independent *t* test for **b**. Two-way ANOVA for **c**, **d**

Then, we further verified the effect of glycogen mobilization on the A1-like and A2-like astrocyte alterations in vitro achieved using GP overexpression (ki-GP) lentiviruses, which was confirmed by immunoblotting (Fig. 2a). RNA sequencing revealed that the mRNA levels of A1-like astrocyte-specific 12 genes were significantly downregulated in ki-GP astrocytes compared with blank vector-treated astrocytes at 72 h following OGD/R (Fig. 2b). In addition, the mRNA levels of A2-like astrocyte-specific 12 genes were upregulated in ki-GP astrocytes at 72 h following OGD/R (Fig. 2b). Next, we determined alterations of A1-like and A2-like astrocytes in different timepoints and found that C3 levels began to be downregulated at 24 h in ki-GP astrocytes compared with blank vector-treated astrocytes after reoxygenation (Fig. 2c). In addition, the S100A10 level began to be upregulated at 12 h in ki-GP astrocytes compared with blank vector-treated astrocytes after OGD/R (Fig. 2d).

### The astrocytic A1/A2 paradigm plays a crucial role in glycogen mobilization-mediated neuroprotection during I/R

**Fig. 3:**
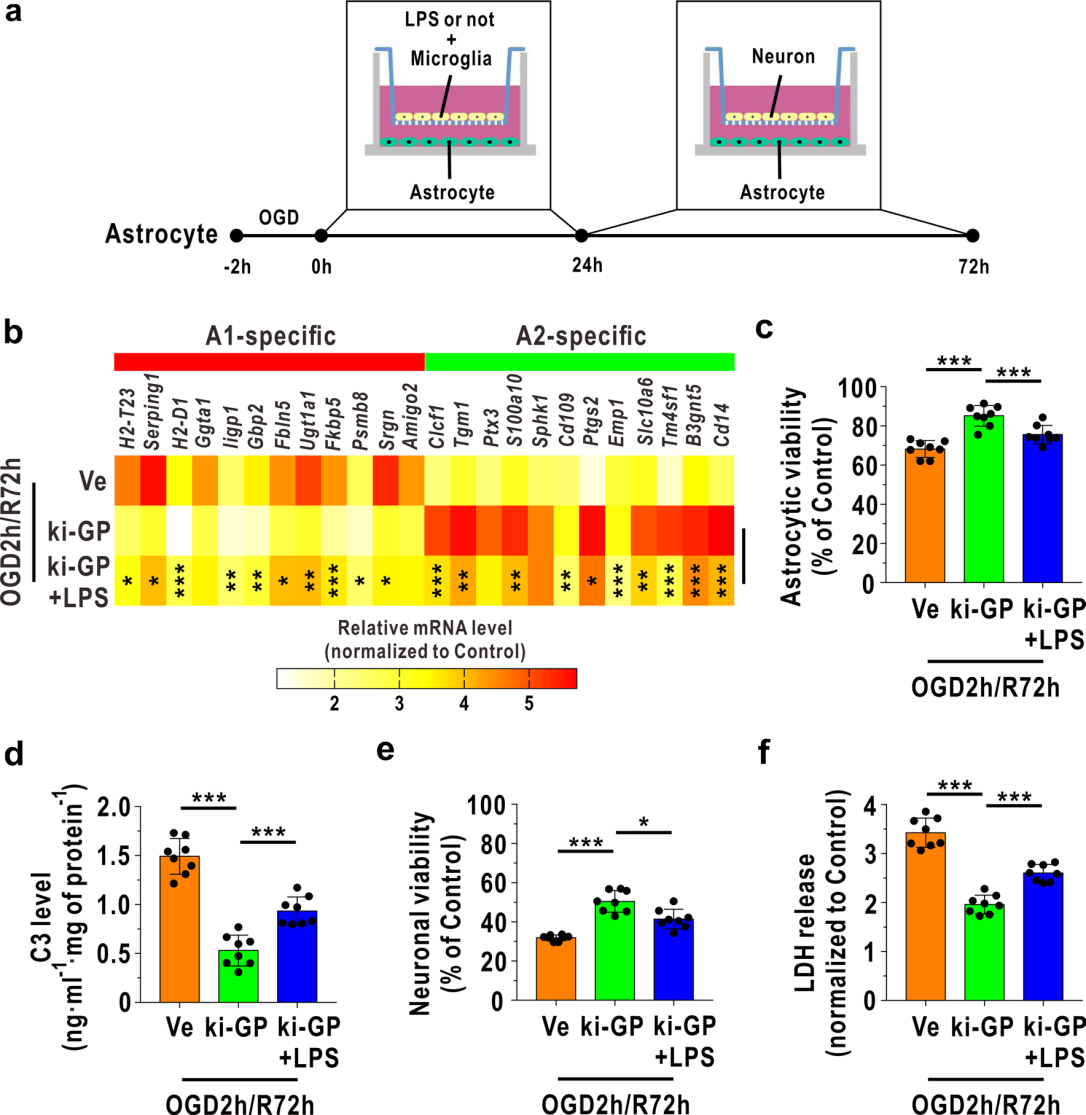
Glycogen mobilization enhances astrocyte and cocultured neuron survival by regulating astrocytic A1/A2 paradigm after OGD/R. **a** Diagram of the astrocyte–microglia coculture model and astrocyte–neuron coculture model. Astrocytes received OGD challenge for 2 h and cocultured with microglia immediately following reoxygenation for 24 h, and then cocultured with neurons at 24 h following reoxygenation for 48 h. The neurons also received the OGD challenge for 2 h and cocultured with astrocytes at 24 h following reoxygenation. LPS was added to astrocyte–microglia cocultures at a concentration of 1:500. **b** Heat map of relative mRNA levels of 12 target genes of A1-like astrocyte and A2-like astrocyte at 72 h following reoxygenation, determined by RNA sequencing (*n* = 4). Statistical analysis was calculated between ki-GP group and ki-GP + LPS group using one-way ANOVA followed by LSD post hoc analysis or Dunnett T3 post hoc analysis. **c** Astrocytic viability at 72 h following OGD/R analyzed by CCK-8 (*n* = 8). **d** C3 levels in the astrocyte–neuron coculture medium at 72 h following OGD/R (*n* = 8). **e** Cocultured neuronal viability at 72 h following OGD/R, as analyzed by the CCK-8 assay (*n* = 8). **f** LDH release in astrocyte–neuron coculture medium at 72 h following OGD/R (*n* = 8). *P < 0.05, **P < 0.01, ***P < 0.001. One-way ANOVA followed by LSD post hoc analysis for **c**, **d**. One-way ANOVA followed by Dunnett T3 post hoc analysis for **e**, **f**

Furthermore, we used an in vitro cell coculture model to investigate whether glycogen mobilization-mediated neuroprotection against cerebral reperfusion injury relied on astrocytic A1/A2 transformation. Astrocytes were cocultured with microglia immediately after OGD/R challenge for 24 h and then with neurons at 24 h after reoxygenation for 48 h (Fig. 3a). The neurons also suffered from OGD challenge and were cultured with astrocytes at 24 h after reoxygenation. A previous study suggested that LPS accelerated microglia to secrete inflammatory factors to enhance A1-like astrocyte formation [16], and LPS could also directly transform astrocytes into the A1-like status via interaction with the toll-like receptor 4 (TLR4) receptor [32]. As shown in Fig. 3b, the mRNA levels of A1-like astrocyte-specific 12 genes were reduced in ki-GP astrocytes and were mostly enhanced when LPS was added to the astrocyte–microglia coculture system. In addition, the mRNA levels of A2-like astrocyte-specific 12 genes were mostly downregulated after LPS treatment at 72 h following reoxygenation (Fig. 3b). Astrocytic viability was increased with GP upregulation but was decreased when LPS was added (Fig. 3c). In addition, C3 in the neuron–astrocyte coculture medium, which was secreted by A1-like astrocytes and was destructive to neurons [16], was decreased when GP was overexpressed and increased with extra LPS treatment at 72 h following reoxygenation (Fig. 3d). Consequently, the viability of cocultured neurons was increased with GP upregulation and decreased when LPS was added to the astrocyte–microglia coculture system (Fig. 3e). LDH release in the neuron–astrocyte coculture was also increased with LPS treatment during OGD/R insult (Fig. 3f).

### Glycogen mobilization regulates astrocytic A1/A2 transformation through the ROS-mediated NF-κB/STAT3 pathway during OGD/R

**Fig. 4:**
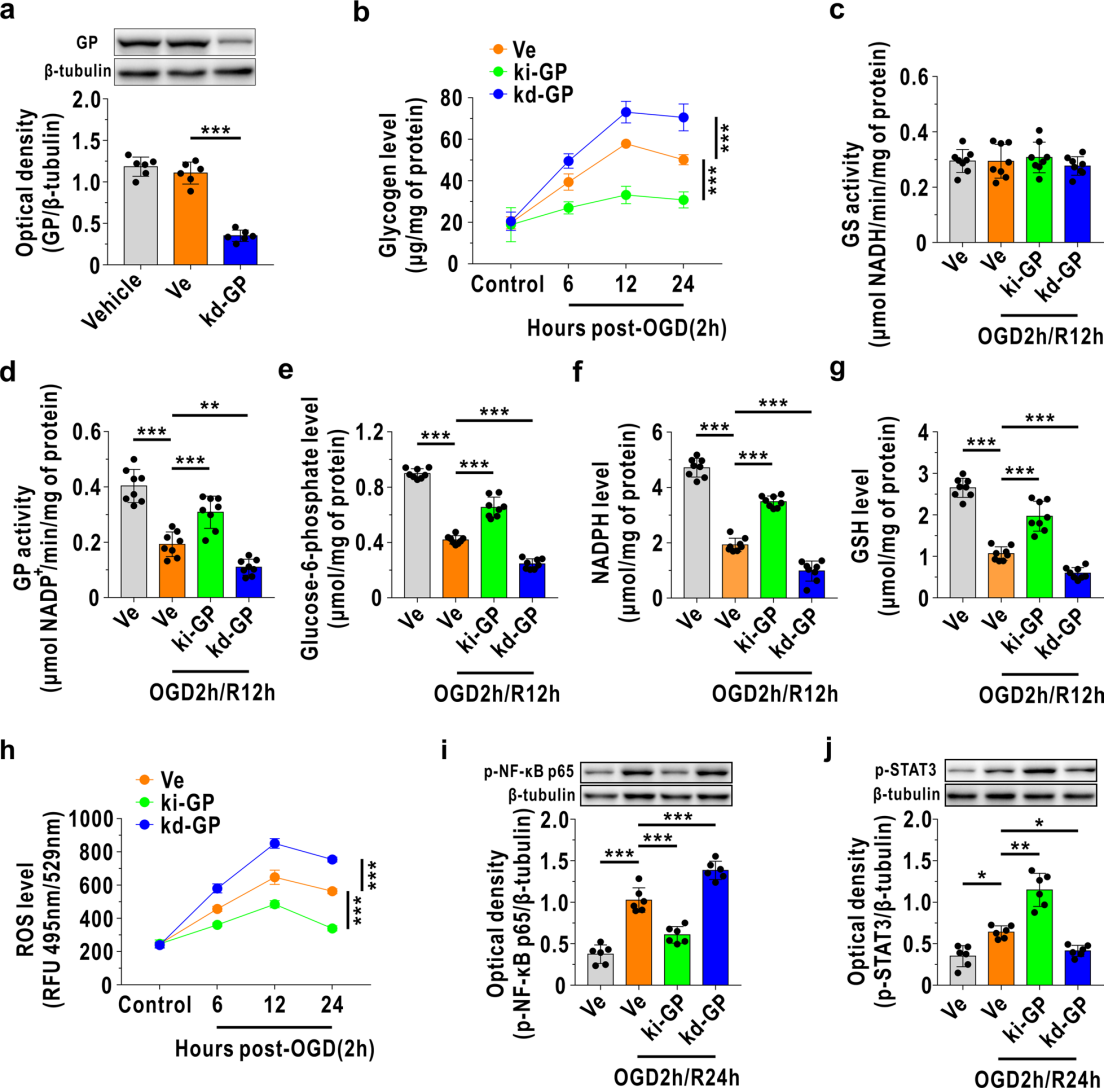
Glycogen mobilization regulates astrocytic A1/A2 paradigm via ROS-mediated NF-κB inhibition and STAT3 activation following OGD/R. **a** Verification of GP knockdown in cultured astrocytes (kd-GP) using immunoblotting (*n* = 6). **b** Glycogen levels in cultured astrocytes at different timepoints before and after OGD/R insult (*n* = 7). **c**, **d** Quantitative analysis of GS (**c**) and GP (**d**) activities at 12 h following reoxygenation (*n* = 8). **e**–**g** Quantitative analysis of glucose-6-phosphate (**e**), NADPH (**f**) and glutathione (GSH, **g**) at 12 h following reoxygenation (*n* = 8). **h** Astrocytic ROS levels at different timepoints before and after OGD/R challenge (*n* = 8). **i**, **j** Protein levels of astrocytic phosphorylated NF-κB p65 (p-NF-κB p65, **i**) and phosphorylated STAT3 (p-STAT3, **j**) at 24 h following reoxygenation (*n* = 6), as measured by immunoblotting. *P < 0.05, **P < 0.01, ***P < 0.001. One-way ANOVA followed by LSD post hoc analysis for **a**, **c**, **d**, **f**, **i**. One-way ANOVA followed by Dunnett T3 post hoc analysis for **e**, **g**, **j**. Two-way ANOVA followed by LSD post hoc analysis for **b**, **h**

Next, the underlying mechanism of glycogen mobilization-induced astrocytic A1/A2 transformation was investigated. We first constructed a GP knockdown model (kd-GP) in cultured astrocytes using CRISPR-Cas9-mediated interference and verified it by immunoblotting (Fig. 4a). As shown in Fig. 4b, we found that reperfusion-induced glycogen accumulation was alleviated in the ki-GP group and was aggravated in the kd-GP group after OGD/R. GS activity was not affected in either the ki-GP model or kd-GP model (Fig. 4c), while GP activity was increased with GP overexpression and decreased with GP knockdown after reoxygenation (Fig. 4d). Glucose-6-phosphate, the degradation product of glycogen, was found to be increased in ki-GP astrocytes compared with blank vector-treated astrocytes and further decreased by GP deficiency (Fig. 4e). In addition, we detected the amounts of NADPH and glutathione and observed that they were both increased upon GP activation and decreased when GP was knocked down after reoxygenation (Fig. 4f, g). GP activation decreased the levels of ROS in cultured astrocytes, which were further increased in the kd-GP model following OGD/R (Fig. 4h). Moreover, NF-κB p65 subunit phosphorylation reflects the activation of NF-κB [33]. Here, we observed that glycogen mobilization inhibited NF-κB activation at 24 h following OGD/R and that this inhibitory impact was blocked once GP was deficient (Fig. 4i). In contrast, STAT3 was activated in the ki-GP group and suppressed in the kd-GP model at 24 h following OGD/R (Fig. 4j).

**Fig. 5:**
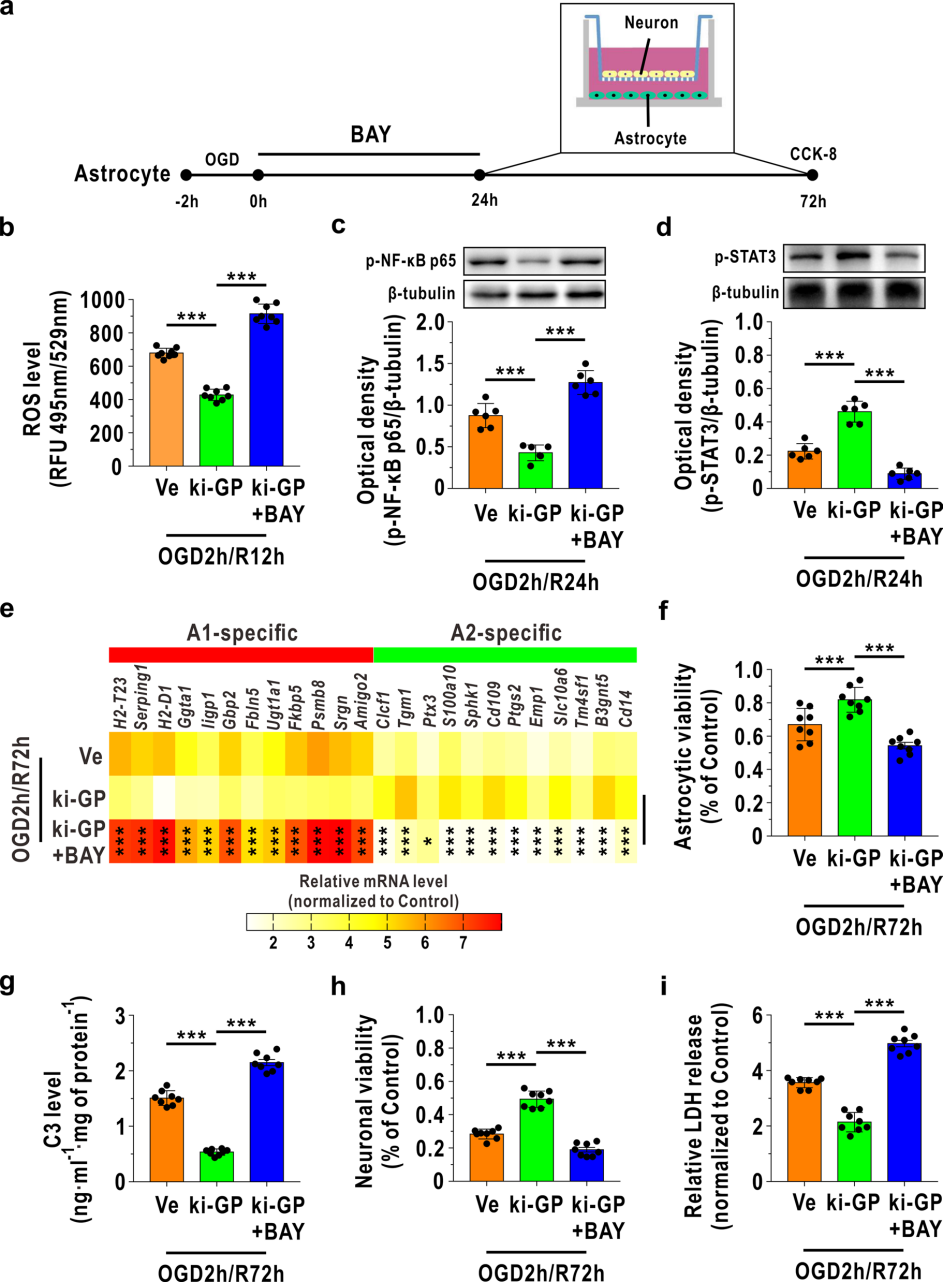
ROS inducer BAY reversed glycogen mobilization-mediated A1/A2 transformation in cultured astrocytes after OGD/R. **a** Astrocytes received the OGD challenge for 2 h, and BAY (10 nM) was added to the astrocytic culture medium immediately following reoxygenation for 24 h. Then, astrocytes were cocultured with neurons at 24 h following reoxygenation for 48 h. The neurons also received the OGD challenge for 2 h and cocultured with astrocytes at 24 h following reoxygenation. **b** Astrocytic ROS levels at 12 h following reoxygenation (*n* = 8). **c**, **d** Protein levels of astrocytic p-NF-κB p65 (**c**) and p-STAT3 (**d**) at 24 h following reoxygenation (*n* = 6), as measured by immunoblotting. **e** Heat map of relative mRNA levels of 12 target genes of A1-like astrocyte and A2-like astrocyte at 72 h following reoxygenation, determined by RNA sequencing (*n* = 4). Statistical analysis was calculated between ki-GP group and ki-GP + BAY group using one-way ANOVA followed by LSD post hoc analysis or Dunnett T3 post hoc analysis. **f** Astrocytic viability at 72 h following OGD/R analyzed by CCK-8 (*n* = 8). **g** C3 levels in the astrocyte–neuron coculture medium at 72 h following OGD/R (*n* = 8). **h** Cocultured neuronal viability at 72 h following OGD/R, as analyzed by the CCK-8 assay (*n* = 8). **i** LDH release in astrocyte–neuron coculture medium at 72 h following OGD/R (*n* = 8). *P < 0.05, ***P < 0.001. One-way ANOVA followed by LSD post hoc analysis for **c**, **d**, **f**, **i**. One-way ANOVA followed by Dunnett T3 post hoc analysis for **b**, **g** and **h**

We then used the ROS inducer BAY to further verify that glycogen mobilization regulates A1/A2 astrocytes via the ROS-mediated NF-κB/STAT3 pathway. As shown in Fig. 5a, astrocytes underwent OGD challenge for 2 h, and BAY was added to the culture medium immediately following reoxygenation for 24 h. Then, the BAY-treated astrocytes were cocultured with neurons at 24 h following OGD/R for 48 h. In addition, the neurons also received OGD challenge for 2 h and were cocultured with astrocytes at 24 h following reoxygenation. The ROS level was upregulated in BAY-treated ki-GP astrocytes compared with ki-GP astrocytes at 12 h following reoxygenation (Fig. 5b). BAY enhanced NF-κB activation and inhibited STAT3 activation at 24 h following reoxygenation (Fig. 5c, d). Consequently, the mRNA levels of A1-like astrocyte-specific 12 genes were increased and the mRNA levels of A2-like astrocyte-specific 12 genes were decreased in BAY-treated ki-GP astrocytes compared with ki-GP astrocytes at 72 h following OGD/R (Fig. 5e). BAY could also damage ki-GP astrocytic viability and induce ki-GP astrocytes to secrete more C3 into coculture medium at 72 h following reoxygenation (Fig. 5f, g). In addition, neuronal survival was decreased, and LDH release in astrocyte–neuron coculture medium was increased when astrocytes were cocultured with BAY-treated ki-GP astrocytes at 72 h following OGD (Fig. 5h, i).

### Glycogen mobilization regulates astrocytic A1/A2 transformation through the ROS-mediated NF-κB/STAT3 pathway during MCAO/R

**Fig. 6:**
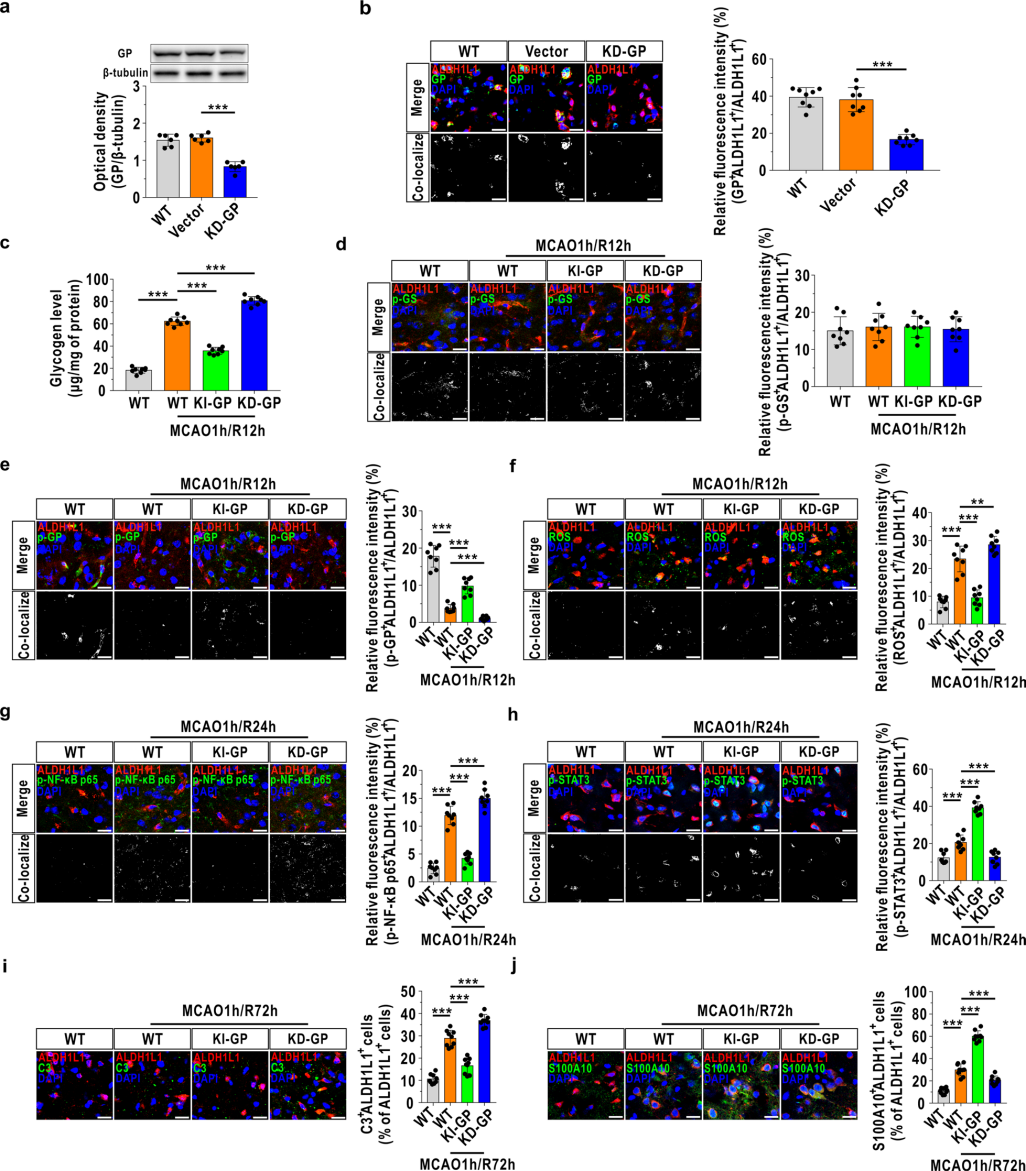
Glycogen mobilization regulates astrocytic A1/A2 paradigm via ROS-mediated NF-κB inhibition and STAT3 activation following MCAO/R. **a** The protein level of GP was analyzed by immunoblotting in frontal cortex area 1 of the astrocyte-specific GP knockdown (KD-GP) mouse brain (*n* = 6). The vector represents mice intracerebroventricularly injected with mock vector AAVs. **b** Left panels: representative images using immunofluorescence staining with antibodies against ALDH1L1 and GP in frontal cortex area 1 of the KD-GP mouse brain (*n* = 8). Right panels: quantitative analysis of the relative fluorescence intensity of GP. The scale bars represent 20 μm. **c** Cerebral glycogen level at 12 h following MCAO injury (*n* = 8). **d**–**h** Coronal images of the cerebral ischemic penumbra following immunofluorescence staining with antibodies against phosphorylated GS (p-GS, **d**), phosphorylated GP (p-GP, **e**), p-NF-κB p65 (**g**) and p-STAT3 (**h**) and an antibody against ALDH1L1 after MCAO/R (*n* = 8). ROS levels were analyzed using the ROS Detection Assay Kit (**f**). The scale bars represent 20 μm. **i**, **j** Coronal images of the cerebral ischemic penumbra following immunofluorescence staining with antibodies against C3 (**i**) and S100A10 (**j**) and an antibody against ALDH1L1 after MCAO/R (*n* = 8). The scale bars represent 20 μm. **P < 0.01, ***P < 0.001. One-way ANOVA followed by LSD post hoc analysis for **c**–**j**. One-way ANOVA followed by Dunnett T3 post hoc analysis for **a**, **b**

The underlying mechanism of glycogen mobilization-mediated astrocytic A1/A2 transformation was further evaluated in the MCAO/R model, which was achieved using astrocyte-specific GP knockdown transgenic mice (KD-GP, Fig. 6a, b). In addition, a newly synthesized antibody toward phosphorylated GP was also constructed (Additional file 1: Fig. S1). Recent studies have revealed that phosphorylated GP is the active form of GP and that phosphorylation inactivates GS [5, 6]. Therefore, the levels of phosphorylated GS and phosphorylated GP represent the activities of GS and GP, respectively. We first observed that the accumulation of glycogen vanished in the KI-GP mice compared with vehicle-injected WT mice and that this decrease was inhibited by GP knockdown after cerebral reperfusion (Fig. 6c). In addition, GP overexpression had no effect on the level of phosphorylated GS (Fig. 6d) but significantly enhanced the level of phosphorylated GP (Fig. 6e). Moreover, ROS levels in astrocytes were decreased in KI-GP mice and were increased following GP knockdown at 12 h following cerebral reperfusion (Fig. 6f). GP overexpression also suppressed NF-κB activation (Fig. 6g) and activated STAT3 at 24 h after reperfusion (Fig. 6h), and these effects were blocked when GP was knocked down. In addition, the observed decrease in A1-like astrocytes and increase in A2-like astrocytes in GP-overexpressing mice were reversed in KD-GP transgenic mice at 72 h after MCAO/R (Fig. 6i, j).

## Discussion

**Fig. 7:**
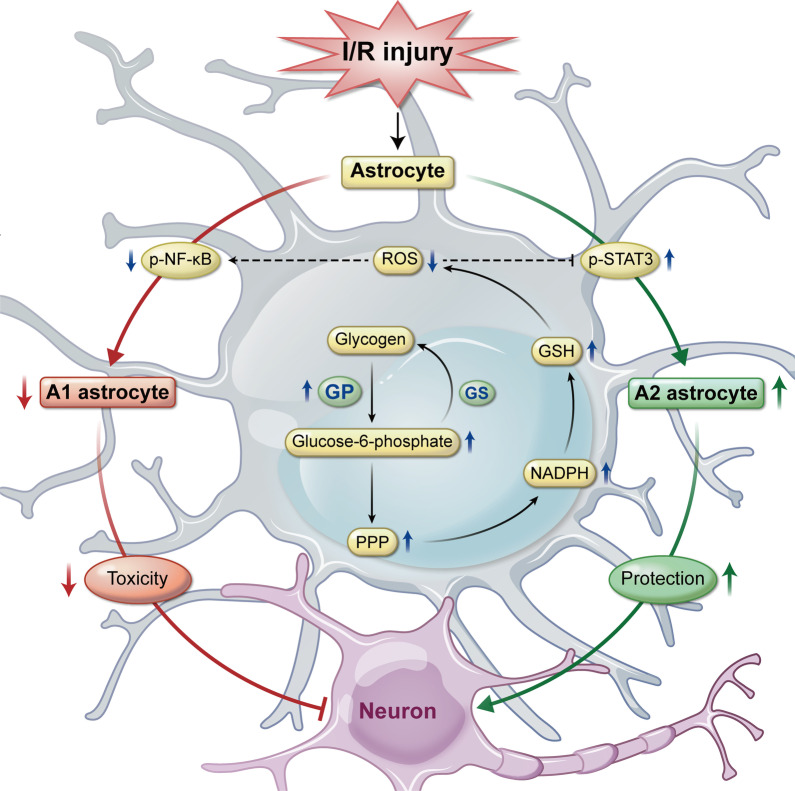
Schematic diagram depicting the mechanism underlying glycogen mobilization-mediated neuroprotection against reperfusion injury after ischemic stroke. *GSH* glutathione

We reveal a previously unsuspected phenomenon in which glycogen mobilization can regulate A1/A2 astrocyte formation to enhance neuronal survival and exert neuroprotective effects against cerebral reperfusion injury. ROS decrease induced NF-κB inhibition and STAT3 activation are upstream pathways of glycogen mobilization-mediated suppression of A1-like astrocytes and acceleration of A2-like astrocytes, respectively. In addition, astrocytic glycogenolysis activation decreased ROS levels via the PPP pathway during recirculation after ischemic stroke (Fig. 7).

For decades, the primary approach for stroke therapy has focused on rescuing ischemic neurons suffering from irreversible insult, but most clinical trials of ischemic stroke have proven that this strategy provides no significant benefits [34]. To broaden therapeutic options, we must reconsider treatment approaches that could contribute to multiple brain cells, particularly astrocytes. Brain glycogen is mostly located in astrocytes but not neurons [15], and astrocytic glycogen was reported to rapidly degrade and provide energy support for neighboring neurons during the energy crisis [7]. Previous studies have suggested that brain glycogen excessively accumulates after cerebral reperfusion. Our group recently uncovered that glycogenolysis but not glycogenesis dysfunction is responsible for reperfusion-induced glycogen accumulation. In addition, enhancing accumulated glycogen degradation exhibits neuroprotective effects on brain reperfusion injury, but the underlying mechanism is unknown, which impedes its clinical therapeutic application. Here, we observed an interesting phenomenon in which glycogenolysis activation transformed astrocytes less into the detrimental type (A1) and more into the beneficial type (A2). This “devil-to-angel” conversion induced by glycogen mobilization benefits the survival of cocultured neurons and the recovery of neurobehavioral function in mice. Thus, glycogen mobilization is likely to act as a coordinator to enhance the resistance of both astrocytes and surrounding neurons rather than simply promoting astrocytic survival when the brain as a whole suffers from reperfusion injury.

The key regulator that transforms astrocytes into A1-like and A2-like subtypes has been far from fully identified, since this concept was introduced [16], and the mechanism by which glycogenolysis activation alters the astrocytic A1/A2 ratio during I/R is unclear. Previous evidence suggests that NF-κB-activated astrocytes release C3 to aggravate brain damage in Alzheimer’s disease [17, 35], and STAT3-ablated astrocytes lose the ability to facilitate neuronal regeneration after trauma [18, 36]. NF-κB acts as a key redox-sensitive transcription factor that can be stimulated by ROS [19], and ROS suppress STAT3 in human epidermoid carcinoma cell lines [20]. In addition, glucose-6-phosphate, the degradation product of glycogenolysis, can enter the PPP to generate endogenous reductive products, including NADPH and glutathione [9, 10]. Hence, it is conceivable, but remains to be formally confirmed, that glycogen mobilization regulates astrocytic A1/A2 conversion via the ROS-mediated NF-κB and STAT3 pathways. To bypass the disturbance of neurons, we generated astrocyte-specific GP overexpression and knockdown mice and demonstrated that the increase in NADPH and glutathione induced by glycogenolysis activation reduces ROS levels, subsequently inhibits NF-κB and upregulates STAT3, suppresses A1-like astrocytes and enhances A2-like astrocytes both in vivo and in vitro during I/R. These results indicate that in the context of ischemia, compromised astrocytes change their roles by stimulating glycogen degradation to boost their energy reserves and fight against oxidative damage. Accordingly, new therapeutic avenues aimed at mobilizing glycogen to harness the detoxification of ROS through PPP are being introduced and will certainly attract more attention in clinical stroke research.

Notably, although there are many studies using the terms “A1” and “A2” to divide astrocytes into harmful and protective astrocytes, a new consensus statement pointed out that this binary division is oversimplified and is not representative of the whole astrocyte transcriptome [37]. This further suggests that astrocyte phenotypes should be defined by a combination of molecular markers and functional readouts [37]. Accordingly, the words “A1-like” not “A1” and “A2-like” not “A2” were adopted in this study to reflect the different statuses of reactive astrocytes determined by 12 gene marker RNA sequencing. In addition, Liddelow’s study suggested that TLR4 was specifically expressed in microglia but not astrocytes in rodent brains, but many other studies provide strong evidence supporting that mouse astrocytes also express TLR4 [38, 39]. In addition, new findings pointed out that the activation of astrocytic TLR4 could enhance astrocyte transformation into the A1-like status [32]. Consequently, the increase in A1-like astrocytes and the decrease in A2-like astrocytes were achieved not only by microglia-secreted inflammatory factors such as C1q, IL-1α and TNF but also by astrocytic TLR4 signaling activation when LPS was added to the astrocyte–microglia coculture system. Another important issue is that Raker’s study suggested that deletion of STAT3 exerts a neuroprotective role in stroke [40], which seems to conflict with the discovery that STAT3 activation enhanced A2-like astrocyte formation after reperfusion in this study. To further verify the relationship between STAT3 and A2-like astrocyte formation, we constructed STAT3 knockdown cultured astrocytes and found that STAT3 ablation decreased the formation of A2-like astrocytes after OGD/R (Additional file 1: Fig. S2). In addition, STAT3 is reported to exert multiple effects in the brain [41, 42]. As a result, STAT3 ablation may activate other intrinsic neuroprotective pathways, notwithstanding the decrease in A2-like astrocyte formation, which comprehensively benefits the outcomes of stroke.

One potential limitation of this study is that we were not able to quantify astrocyte-specific small molecule metabolites in vivo. Double-labeling immunofluorescence allows us to detect the activity of enzymes but is not suitable for substances, such as glucose-6-phosphate, NADPH and glutathione. To solve this problem, we analyzed the alterations of these metabolites in cultured astrocytes during OGD/R to indirectly reflect the changes in vivo. New precise methods are urgently needed to analyze astrocyte-specific metabolites in vivo in the future. Another limitation of this study is that BAY, a mitochondrial complex I inhibitor, exhibits a variety of detrimental cellular events which not only increase the ROS production but also induce a decrease in total cellular ATP, the activation of AMP-activated protein kinase (AMPK) signaling and the partial depolarization of the mitochondrial membrane potential, and consequently is cytotoxic to cancer cells [24, 43, 44]. Whether BAY also has multiple effects on neural cells has not been reported, but there is a possibility that BAY reversed glycogen mobilization-mediated A1/A2 transformation in cultured astrocytes after OGD/R, which was not attributed to the increase in ROS but might be due to the other cellular events mentioned above. To our knowledge, there is no ROS-specific inducer that is restricted to increasing ROS production and has no impacts on the other cellular events. Accordingly, BAY cannot be replaced by other ROS-specific inducers at present. Further studies can focus on this issue to invent ROS-specific inducers in the future.

## Conclusions

Collectively, our findings indicate that ROS-mediated NF-κB suppression and STAT3 activation underlie glycogen mobilization-induced astrocytic A1/A2 transformation after cerebral reperfusion. Glycogen mobilization enhances NADPH and glutathione levels via the PPP pathway to decrease ROS levels after I/R. This study established a direct link between glycogen mobilization and the neuroprotective effect against cerebral reperfusion injury after ischemic stroke.

## Supplementary Information

**Additional file 1: Fig. S1.** Identification of the phosphorylated GP (p-GP) antibody. (a) The absorbance at 450 nm of the p-GP antibody toward phosphorylated antigen and unphosphorylated antigen using indirect ELISA with an increasing dilution ratio (n = 4). (b) Recognition of p-GP by the p-GP antibody in coronal slices of mouse frontal cortex area 1 by immunofluorescence (dilution ratio of 1:500). The scale bar represents 20 μm. **Fig. S2.** STAT3 ablation suppresses the A2-like astrocyte formation following OGD/R. (a) Verification of STAT3 knockdown cultured astrocytes (kd-STAT3) using immunoblotting (n = 6). Vehicle represents unaffected astrocytes. Ve represents astrocytes affected by blank lentiviruses. (b) Protein levels of phosphorylated STAT3 (p-STAT3) determined by immunoblotting (n = 6). (c) Heat map of relative mRNA levels of 12 target genes of A2-like astrocyte at 72 h following reoxygenation, determined by RNA sequencing (n = 4).^*^P<0.05,^**^P< 0.01, ^***^P < 0.001. One-way ANOVA followed by LSD post hoc analysis for (a) and (b). Independent t-test for (c). **Fig. S3.** The original immunofluorescence images for Fig. 1. The full uncropped immunofluorescence images for Fig. 1a (a), Fig. 1c (b) and Fig. 1d (c). The scale bars represent 20 μm. **Fig. S4.** The original immunofluorescence images for Fig. 6. The full uncropped immunofluorescence images for Fig. 6b (a), Fig. 6d (b), Fig. 6e (c), Fig. 6f (d), Fig. 6g (e), Fig. 6h (f), Fig. 6i (g) and Fig. 6j (h). The scale bars represent 20 μm. **Fig. S5.** The original immunoblotting images for Fig. 4i. The left panel is the uncropped immunoblotting image for p-NF-κB p65, and the right panel is the corresponding uncropped image for β-tubulin. **Fig. S6.** The original immunoblotting images for Fig. 5c. The left panel is the uncropped immunoblotting image for p-NF-κB p65, and the right panel is the corresponding uncropped β-tubulin image. **Table S1.** Sources of the antibodies used in this study.

## Data Availability

The data sets during the current study are available from the corresponding author on reasonable request.

## Abbreviations

I/R: Ischemia/reperfusion
GP: Glycogen phosphorylase
NF-κB: Nuclear transcription factor-κB
GS: Glycogen synthase
PPP: Pentose phosphate pathway
ROS: Reactive oxygen species
BAY: BAY 87-2243
MCAO/R: Middle cerebral artery occlusion/reperfusion
OGD/R: Oxygen–glucose deprivation/reoxygenation
AAV: Adeno-associated virus
TTC: Triphenyl tetrazolium chloride
UDPG: Uridine diphosphate glucose
LDH: Lactate dehydrogenase
LPS: Lipopolysaccharide
WT: Wild-type
