# Supplementary material for: Astrocytic A1/A2 paradigm participates in glycogen mobilization mediated neuroprotection on reperfusion injury after ischemic stroke
Source: J Neuroinflammation. 2021 Oct 13;18:230. doi: 10.1186/s12974-021-02284-y (PMC8513339; doi:10.1186/s12974-021-02284-y)
Supplement: Supplementary file 1 — Additional file 1: Fig. S1. Identification of the phosphorylated GP (p-GP) antibody. (a) The absorbance at 450 nm of the p-GP antibody toward phosphorylated antigen and unphosphorylated antigen using indirect ELISA with an increasing dilution ratio (n = 4). (b) Recognition of p-GP by the p-GP antibody in coronal slices of mouse frontal cortex area 1 by immunofluorescence (dilution ratio of 1:500). The scale bar represents 20 μm. Fig. S2. STAT3 ablation suppresses the A2-like astrocyte formation following OGD/R. (a) Verification of STAT3 knockdown cultured astrocytes (kd-STAT3) using immunoblotting (n = 6). Vehicle represents unaffected astrocytes. Ve represents astrocytes affected by blank lentiviruses. (b) Protein levels of phosphorylated STAT3 (p-STAT3) determined by immunoblotting (n = 6). (c) Heat map of relative mRNA levels of 12 target genes of A2-like astrocyte at 72 h following reoxygenation, determined by RNA sequencing (n = 4).*P<0.05,**P< 0.01, ***P < 0.001. One-way ANOVA followed by LSD post hoc analysis for (a) and (b). Independent t-test for (c). Fig. S3. The original immunofluorescence images for Fig. 1. The full uncropped immunofluorescence images for Fig. 1a (a), Fig. 1c (b) and Fig. 1d (c). The scale bars represent 20 μm. Fig. S4. The original immunofluorescence images for Fig. 6. The full uncropped immunofluorescence images for Fig. 6b (a), Fig. 6d (b), Fig. 6e (c), Fig. 6f (d), Fig. 6g (e), Fig. 6h (f), Fig. 6i (g) and Fig. 6j (h). The scale bars represent 20 μm. Fig. S5. The original immunoblotting images for Fig. 4i. The left panel is the uncropped immunoblotting image for p-NF-κB p65, and the right panel is the corresponding uncropped image for β-tubulin. Fig. S6. The original immunoblotting images for Fig. 5c. The left panel is the uncropped immunoblotting image for p-NF-κB p65, and the right panel is the corresponding uncropped β-tubulin image. Table S1. Sources of the antibodies used in this study. [file 12974_2021_2284_MOESM1_ESM.docx]

**Supplemental Material**


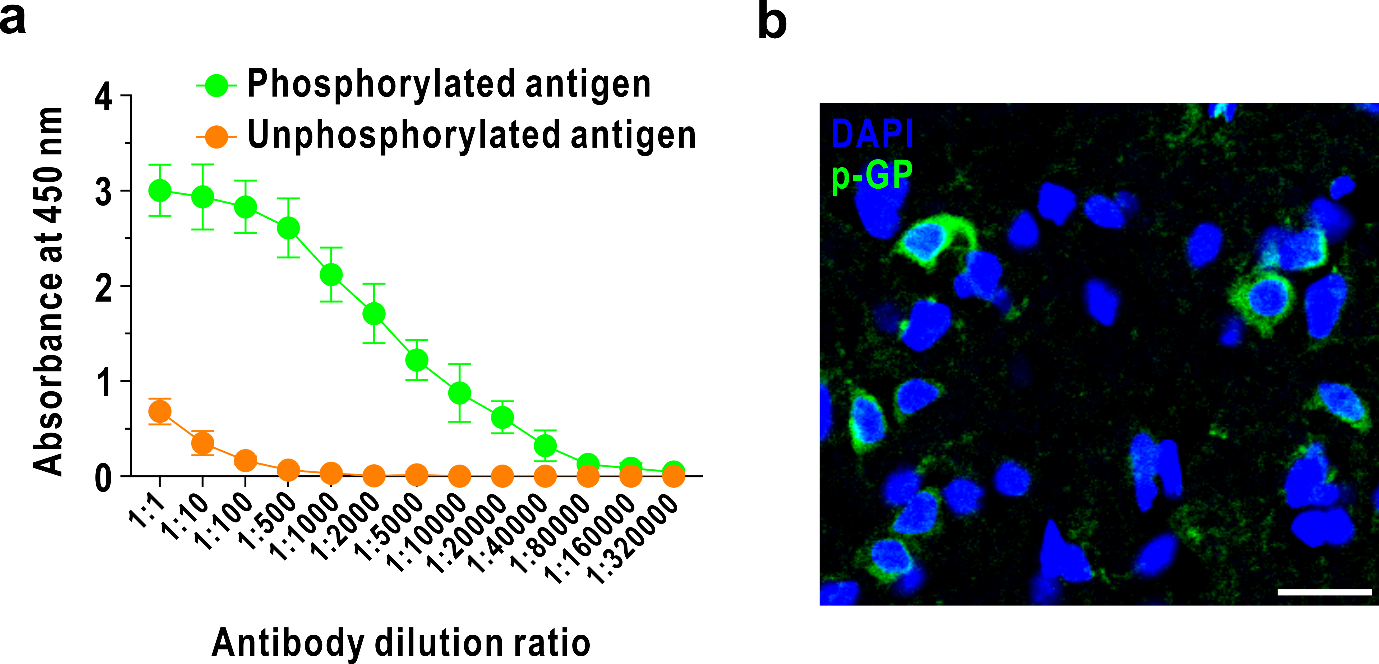


**Fig. S1.** Identification of the phosphorylated GP (p-GP) antibody. (a) The absorbance at 450 nm of the p-GP antibody toward phosphorylated antigen and unphosphorylated antigen using indirect ELISA with an increasing dilution ratio (n = 4). (b) Recognition of p-GP by the p-GP antibody in coronal slices of mouse frontal cortex area 1 by immunofluorescence (dilution ratio of 1:500). The scale bar represents 20 μm.

**
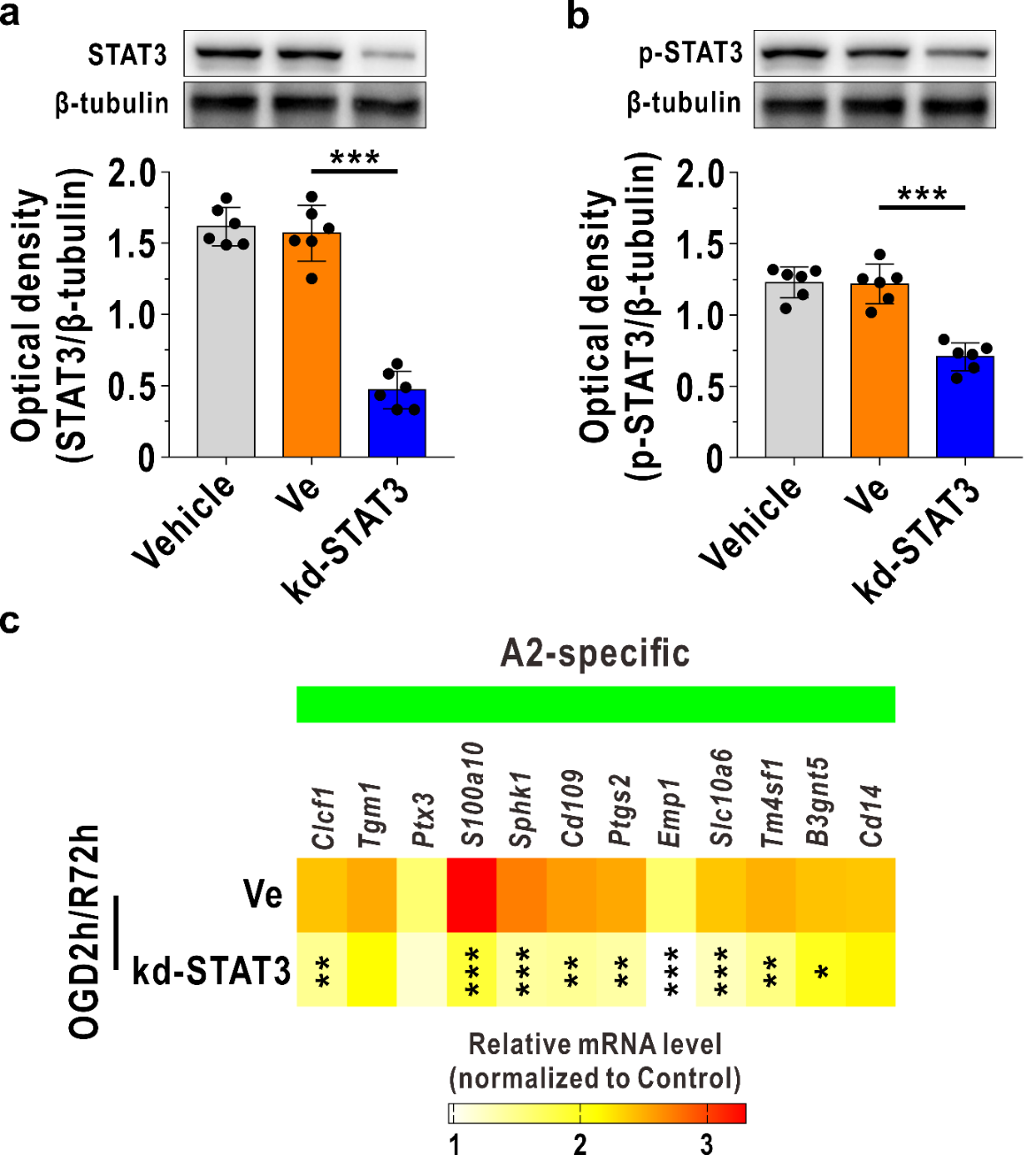
**

**Fig. S2.** STAT3 ablation suppresses the A2-like astrocyte formation following OGD/R. (a) Verification of STAT3 knockdown cultured astrocytes (kd-STAT3) using immunoblotting (n = 6). Vehicle represents unaffected astrocytes. Ve represents astrocytes affected by blank lentiviruses. (b) Protein levels of phosphorylated STAT3 (p-STAT3) determined by immunoblotting (n = 6). (c) Heat map of relative mRNA levels of 12 target genes of A2-like astrocyte at 72 h following reoxygenation, determined by RNA sequencing (n = 4). ^*^*P* < 0.05, ^**^*P* < 0.01, ^***^*P* < 0.001. One-way ANOVA followed by LSD post hoc analysis for (a) and (b). Independent t-test for (c).

**
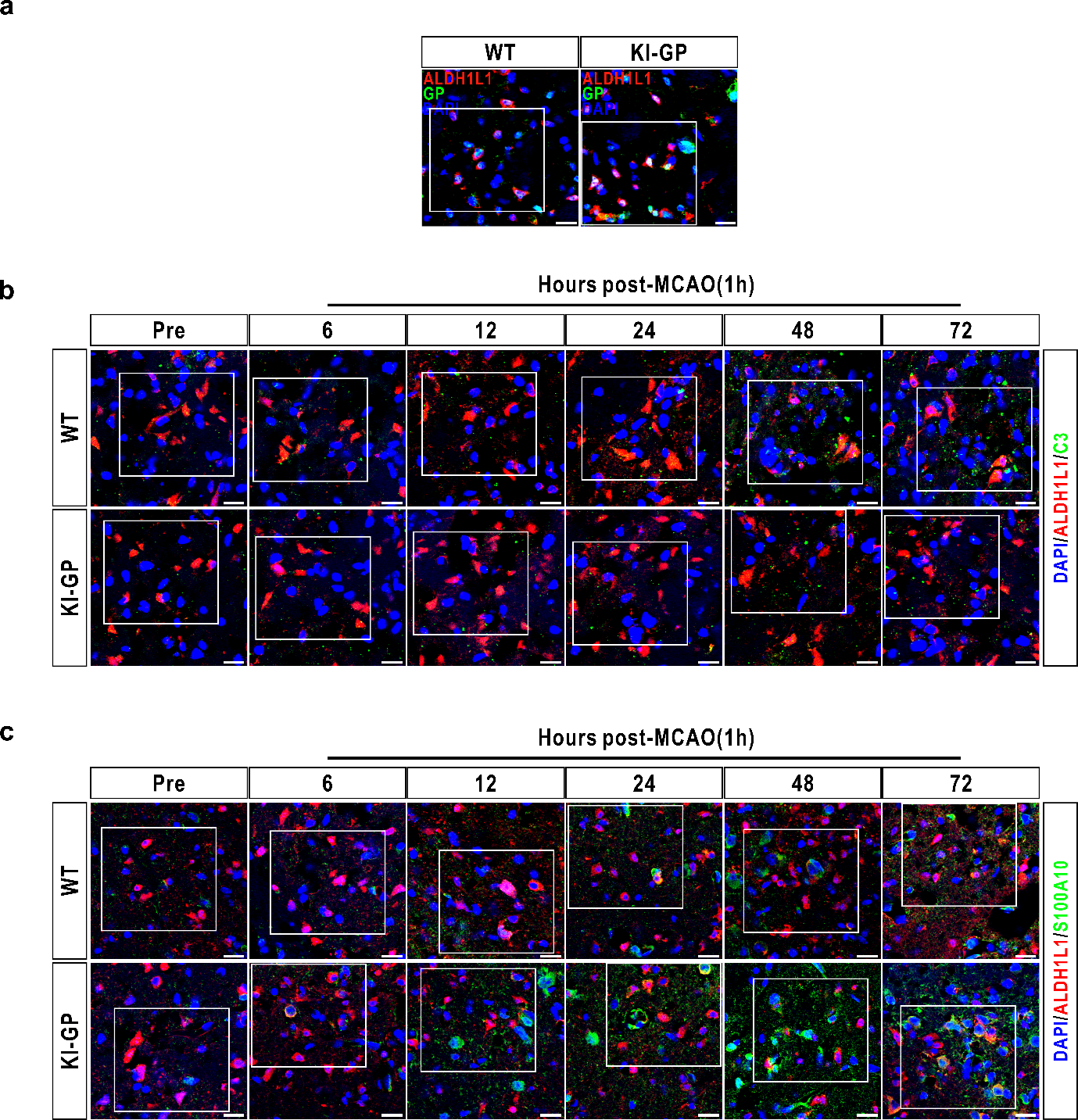
Fig. S3.** The original immunofluorescence images for Fig. 1. The full uncropped immunofluorescence images for Fig. 1a (a), Fig. 1c (b) and Fig. 1d (c). The scale bars represent 20 μm.


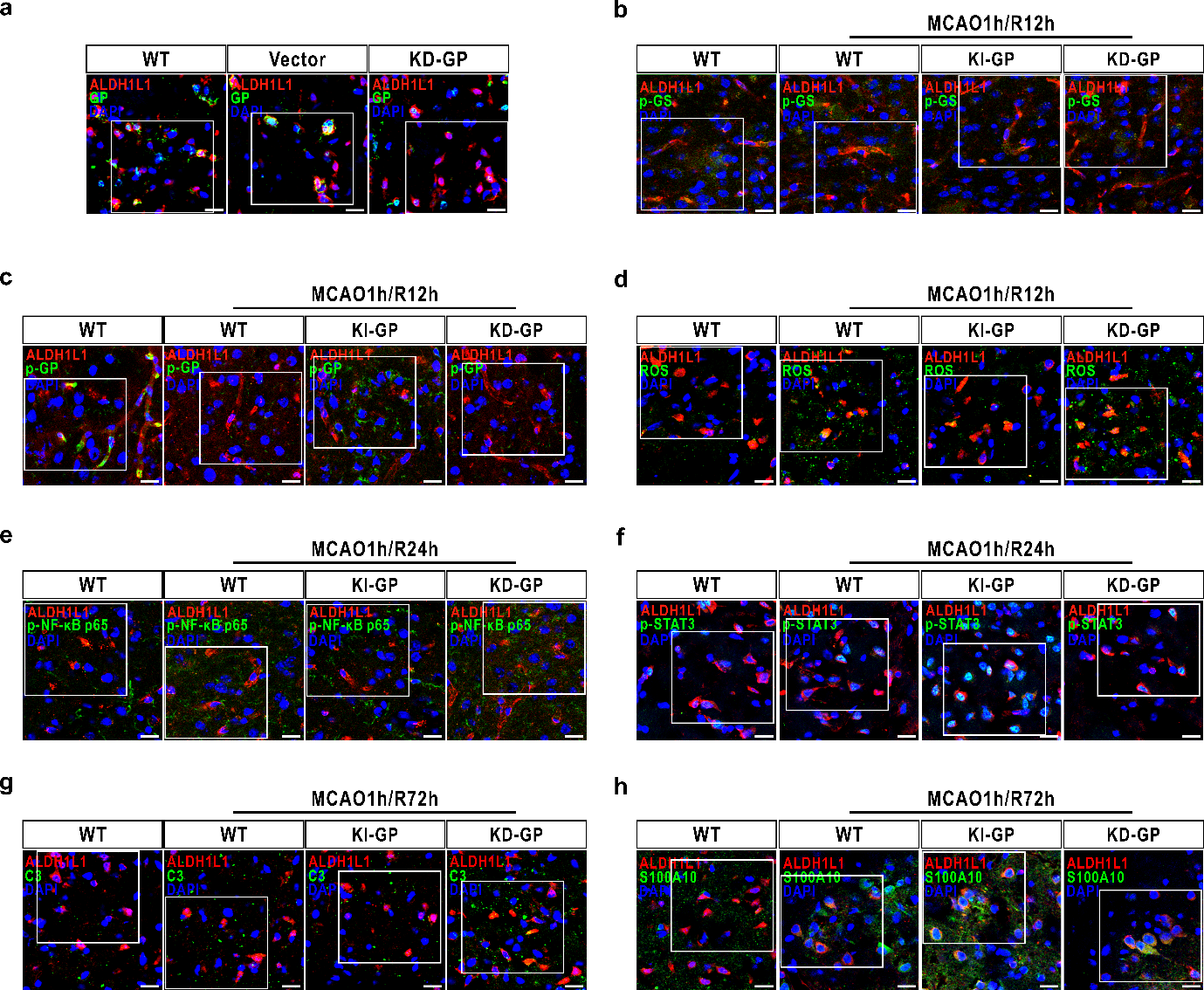


**Fig. S4.** The original immunofluorescence images for Fig. 6. The full uncropped immunofluorescence images for Fig. 6b (a), Fig. 6d (b), Fig. 6e (c), Fig. 6f (d), Fig. 6g (e), Fig. 6h (f), Fig. 6i (g) and Fig. 6j (h). The scale bars represent 20 μm.


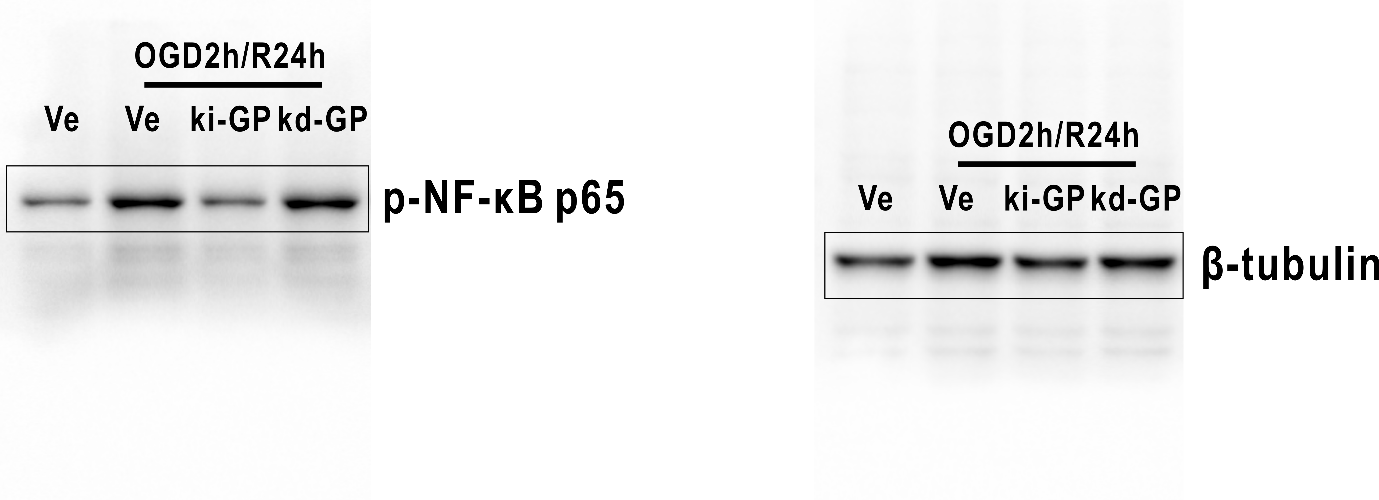


**Fig. S5.** The original immunoblotting images for Fig. 4i. The left panel is the uncropped immunoblotting image for p-NF-κB p65, and the right panel is the corresponding uncropped image for β-tubulin.


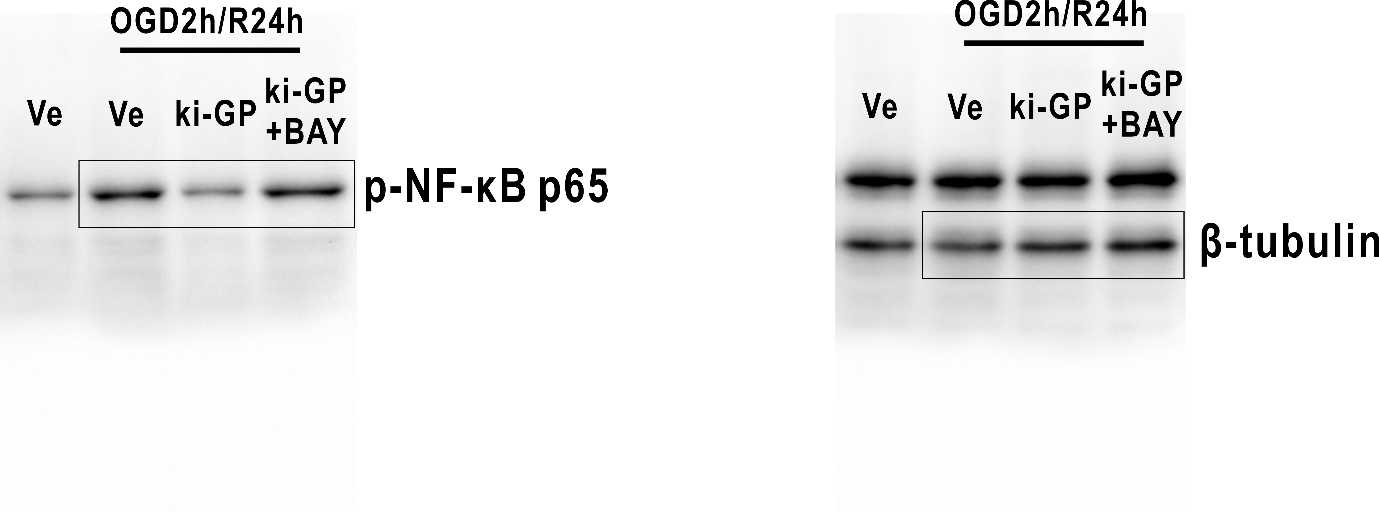


**Fig. S6.** The original immunoblotting images for Fig. 5c. The left panel is the uncropped immunoblotting image for p-NF-κB p65, and the right panel is the corresponding uncropped β-tubulin image.

**Table S1.** Sources of the antibodies used in this study.

| **Target of antibody** | **Source** | **Application** | **Dilution** | **Identifier** |
| --- | --- | --- | --- | --- |
| GP | Abcam | IB | 1:1000 | ab154969 |
| GP | Atlas Antibodies | IF | 1:100 | HPA031067 |
| Phosphorylated GS (Ser641) | Cell Signaling Technology | IF | 1:200 | 47043 |
| β-tubulin | Cell Signaling Technology | IB | 1:1000 | 2128 |
| ALDH1L1 | Abcam | IF | 1:100 | ab177463 |
| C3 | Abcam | IB, IF | 1:1000, 1:100 | ab181147 |
| S100A10 | Thermo Fisher Scientific | IB, IF | 1:1000, 1:100 | PA5–95505 |
| Phosphorylated NF-κB p65 (S276) | Abcam | IB, IF | 1:1000, 1:100 | ab183559 |
| Phosphorylated STAT3 (Y705) | Abcam | IB, IF | 1:2000, 1:200 | ab76315 |
| Mouse IgG H&L (Fluor 488) | Thermo Fisher Scientific | IF | 1:500 | A-11029 |
| Mouse IgG H&L (Fluor 594) | Thermo Fisher Scientific | IF | 1:500 | A32742 |
| Rabbit IgG H&L (Fluor 488) | Thermo Fisher Scientific | IF | 1:500 | A32731 |
| Rabbit IgG H&L (Fluor 594) | Thermo Fisher Scientific | IF | 1:500 | R37117 |
| Rabbit IgG H&L (HRP) | Abcam | IB | 1:5000 | ab6721 |
| Mouse IgG H&L (HRP) | Abcam | IB | 1:5000 | ab6789 |
| Phosphorylated GP (Ser14) | GeneCreate Biotech | IF | 1:500 | Customized antibody |

IB: immunoblotting; IF: immunofluorescence.
